# Comprehensive metabolomic profiling of *Angiostrongylus cantonensis* across its life cycle reveals developmental and host-associated metabolic signatures

**DOI:** 10.1186/s40249-026-01509-7

**Published:** 2026-09-30

**Authors:** Yue Hu, Bingcheng Yu, Rongjian Zhan, Tao Zhang, Yiyang Zhang, Yunfei Zhou, Hang Wei, Zhiyue Lv

**Affiliations:** 1 Key Laboratory of Tropical Disease Control (Sun Yat-sen University), Ministry of Education, Guangzhou, Guangdong China; 2 Provincial Engineering Technology Research Center for Biological Vector Control, Guangzhou, Guangdong China; 3 Department of Urology, Sun Yat-sen Memorial Hospital, Sun Yat-sen University, Guangzhou, Guangdong China; 4 Department of Otorhinolaryngology, The First Affiliated Hospital of Sun Yat-sen University, Guangzhou, Guangdong China; 5 Department of Rheumatology and Clinical Immunology, The First Affiliated Hospital of Sun Yat-sen University, Guangzhou, Guangdong China; 6 Department of Endocrinology, Sun Yat-sen Memorial Hospital, Sun Yat-sen University, Guangzhou, Guangdong China; 7 National Health Commission of the People’s Republic of China (NHC) Key Laboratory of Tropical Disease Control, Hainan Medical University, Haikou, Hainan China

**Keywords:** *Angiostrongylus cantonensis*, Metabolomics, Developmental stage, Host-associated putative metabolite, Metabolic reprogramming

## Abstract

**Background:**

*Angiostrongylus cantonensis* is a zoonotic nematode that causes eosinophilic meningitis in humans. Although its life cycle encompasses multiple developmental stages and hosts, the metabolic changes underlying its development and host interactions remain poorly understood. This study aimed to characterize the dynamic metabolomic profiles of *A. cantonensis* across its entire life cycle and to identify metabolic signatures associated with development and different host environments.

**Methods:**

Untargeted metabolomics using liquid chromatography-mass spectrometry was performed on five key life cycle stages of *A. cantonensis*: first-stage larvae, third-stage larvae (L3), mouse-derived and rat-derived fourth-stage larvae (L4), fifth-stage larvae, and female and male adults. Multivariate statistical analyses, including principal component analysis, partial least squares-discriminatory analysis, and orthogonal partial least squares-discriminatory analysis, were employed alongside univariate tests, random forest (RF) modeling, and pathway enrichment analysis to detect differential metabolites and key pathways.

**Results:**

A total of 4517 and 2613 ion features were detected in positive and negative electrospray ionization modes, respectively. Metabolic profiles shifted markedly, especially during the L3-to-L4 transition, with over 2000 differentially regulated features (*P* < 0.05). A total of 127 unique putatively annotated compounds were identified, including fatty acyls, purine nucleosides, and steroids, among others. Putative metabolites such as hypoxanthine, inosine, and prostaglandins were upregulated at the fourth larval stage, whereas xanthosine peaked in female adults (*P* < 0.05). RF models identified daidzin and docosapentaenoic acid as key discriminators between L3 and L4, and 4-phenylbutyric acid as a discriminator between rat-derived and mouse-derived L4. Pathway analysis revealed that vitamin B6 metabolism, thiamine metabolism, and nicotinate and nicotinamide metabolism were significantly altered during L3-to-L4 transition and were also host-associated (*P* < 0.01).

**Conclusions:**

This comprehensive metabolomic analysis of *A. cantonensis* provides a detailed map of its metabolic landscape across the life cycle. The findings reveal profound metabolic reprogramming during parasite establishment in the definitive host and identify host-associated metabolic signatures. The key putatively annotated compounds and pathways offer new insights into parasite biology, host-parasite interactions, and potential targets for diagnostics and therapeutics against angiostrongyliasis. Importantly, the functional interpretations presented herein are hypothesis-generating and require experimental validation through targeted functional studies.

**Graphical Abstract:**

**Figure:**
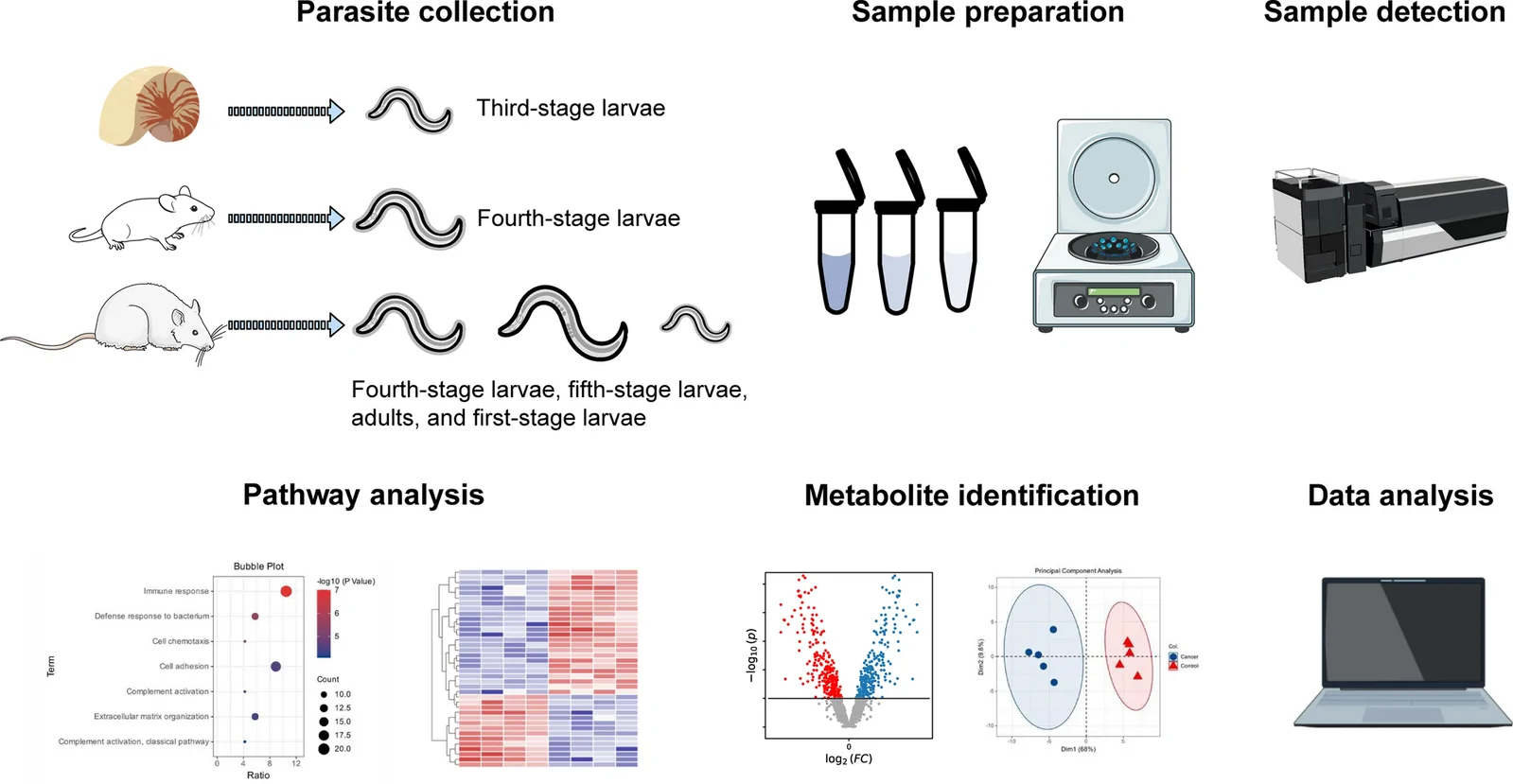

**Supplementary Information:**

The online version contains supplementary material available at https://doi.org/10.1186/s40249-026-01509-7.

## Background

*Angiostrongylus cantonensis* is a food-borne nematode with a short generation time, a straightforward life cycle, ease of laboratory maintenance, and available genomic resources. These attributes establish it as an excellent model organism for diverse biological research applications [1, 2]. Its life cycle involves both intermediate and definitive hosts. Adult worms reside in the pulmonary arteries of rats, where females release eggs that hatch into first-stage larvae (L1). These larvae penetrate alveolar walls, migrate up the respiratory tract, are swallowed, and subsequently are excreted in feces. Terrestrial and aquatic mollusks, including snails and slugs, serve as intermediate hosts, acquiring L1 from contaminated environments. Within these mollusks, larvae undergo two molts to become third-stage larvae (L3), the infective stage for definitive hosts. Rats become infected by consuming infected mollusks or paratenic hosts harboring L3. After ingestion, the parasites penetrate the intestinal wall and migrate via the bloodstream to the central nervous system (CNS), where they develop into fourth-stage (L4) and fifth-stage larvae (L5). Approximately 4 weeks post-infection, young adults migrate to the pulmonary arteries, mature, mate, and produce eggs, thereby completing the cycle. Humans are accidental, non-permissive hosts, becoming infected through ingestion of L3. The larvae migrate preferentially to the brain, causing mechanical injury and inflammatory responses that manifest clinically as eosinophilic meningitis or meningoencephalitis. The disease is characterized by rapid onset and poor prognosis, posing a significant threat to affected individuals [3]. However, the mechanisms of angiostrongyliasis are multifactorial and incompletely understood. Metabolomics offers a powerful approach to probe the physiological state, adaptability, and environmental responses of *A. cantonensis*, potentially providing new insights for disease research.

Metabolomics relies on high-throughput technologies to simultaneously detect and identify numerous metabolites, including small organic molecules, metabolic intermediates, and end products. Current methodologies primarily include nuclear magnetic resonance (NMR), mass spectrometry (MS), infrared spectroscopy (IR), and chromatography-based techniques such as gas chromatography-MS (GC-MS) and liquid chromatography-MS (LC-MS) [4]. LC-MS and GC-MS are widely used to identify and quantify metabolites with diverse chemical properties, offering high sensitivity, strong specificity, and rapid analysis [5].

As an emerging omics discipline, metabolomics detects downstream products of genes and proteins, providing insights into organismal pathological states. Metabolites are relatively limited in number and structurally simpler than genes or proteins, and metabolomics does not require complete genome sequences or extensive expression sequence tag databases. Its applications are growing in early disease diagnosis, drug target discovery [6], and metabolic studies in both animals and plants [7, 8]. In parasitology, metabolomics has been applied to investigate host metabolic responses to parasites [9], identify diagnostic biomarkers [10], and elucidate drug mechanisms against parasitic diseases [11].

Despite these advances, metabolomics research on *A. cantonensis* remains limited. This study represents the comprehensive metabolomic analysis of this parasite from different host sources. We investigated metabolic changes across various developmental stages (L1, L3, L4, L5, and adults) to deepen our understanding of its life cycle. The selected stages represent key biological transitions in the life cycle of *A. cantonensis*. The L1 represents the environmental, free-living form; L3 is the infective stage that encounters the definitive host digestive tract; the L3-to-L4 transition is a critical bottleneck for CNS establishment; and progression from L4 to L5 and adults involves migration from the CNS to the pulmonary arteries and sexual maturation. These transitions are associated with major morphological, physiological, and metabolic changes, making them ideal focal points for metabolomic investigation. This research contributes to further exploration of the pathological and physiological mechanisms underlying disease onset and progression. Additionally, high-throughput screening of specific differential putative metabolites may enable early diagnosis and targeted treatment of angiostrongyliasis, providing new opportunities for drug target development.

## Methods

### Animals and parasite

The complete life cycle of *A. cantonensis* was maintained in *Biomphalaria glabrata* as intermediate host and rats as the definitive host. Female BALB/c mice and Sprague Dawley (SD) rats, approximately 6–8 weeks old, were purchased from Charles River Laboratories (Beijing, China) and housed in plastic cages. A total of 15 BALB/c mice and 45 SD rats were used. Sample size was determined by power analysis (power = 0.8, *α* = 0.05) based on preliminary worm recovery data, indicating a minimum of 15 animals per group. After acclimatization, each mouse and rat was infected intragastrically with 30 and 100 *A. cantonensis* L3, respectively.

### Sample collection

A schematic workflow of the experimental design is presented in Additional file 1. L1 were isolated from feces of *A. cantonensis*-infected rats at 42 days post-infection (dpi). Feces were allowed to sediment in dechlorinated water for at least 4 h, and the sediment containing L1 was collected. L3 were obtained from infected *B. glabrata* by digesting minced snail tissue in a pepsin-HCl solution incubated for 40 min at 37 °C. Digestion was terminated by adding an appropriate volume of PBS, and L3 were then collected under a stereoscopic microscope (SZ650, Cnoptec, Chongqing, China). L4 were recovered from whole brains of mice and rats 3 weeks after infection, while L5 were harvested from whole brains of rats at 28 dpi. Six weeks after infection, female and male adults were collected from the pulmonary artery of rats. All collected worms were washed three times with PBS and stored at −80 °C until analysis. For each life cycle stage, ten biological replicates were generated to account for potential parasite batch variations. Each biological replicate was normalized to approximately 5 mg wet weight prior to extraction: for L1 and L3, this corresponded to pooling 8000–10,000 L1 or 800–1000 L3; for L4, L5, and adults, 2–5 individual worms were used per replicate because a single worm provided sufficient biomass for detection. All replicates from the same stage were pooled from multiple donor animals to minimize individual host variation.

### Metabolite extraction

Samples from each life cycle stage were added to 1 ml of prechilled methanol/water (1 : 1, *v *: *v*) containing magnetic beads and thoroughly homogenized in an ice bath. The suspension was centrifuged at 16,000 × *g* for 10 min at 4 °C, and the supernatant was transferred into an Eppendorf tube and then subjected to vacuum freeze-drying to obtain an aqueous extract. Subsequently, 1 ml of precooled dichloromethane/methanol (3:1, *v*:*v*) was added to the sediment, homogenized again at low temperature, and centrifuged under the same conditions. The supernatant was transferred to a new Eppendorf tube and evaporated to obtain an organic extract. Both aqueous and organic extracts were redissolved in 100 μl of precooled acetonitrile/water (4 : 1, *v *: *v*) after drying and centrifuged for 10 min at 15,000 × *g* at 4 °C to remove particulates. Approximately 80 μl of supernatant was transferred to a sampling vial. Post-acquisition signal normalization was performed using total ion current normalization to correct for injection volume and instrument response variations. Quality control (QC) samples were prepared by pooling equal volumes of all test specimens into the vial to ensure analytical stability and reproducibility. Solvent blank control was also prepared and analyzed following the same workflow.

### Instrumental conditions

Metabolite separation and detection were performed using an Ultimate 3000-Q Exactive High-Performance Liquid Chromatography (UHPLC) system (Thermo Fisher Scientific, Waltham, MA, USA). Chromatographic separation was achieved on a HSS Hilic column (2.1 × 100 mm, 1.9 μm; Waters, Milford, MA, USA) with autosampler and column temperatures maintained at 4 °C and 40 °C, respectively. The flow rate was 0.4 ml/min, and the sample injection volume was 5 μl. Mobile phase A consisted of 95% acetonitrile and 5% Milli-Q water, while mobile phase B consisted of 50% acetonitrile and 50% Milli-Q water, both containing 10 mmol/L ammonium acetate. Gradient elution was performed as follows: 0 min, 1% B; 1 min, 1% B; 13 min, 100% B; 15 min, 100% B; 15.1 min, 1% B; 18 min, 1% B.

MS was conducted using an electrospray ionization (ESI) source operated in positive and negative ion modes, respectively. The experimental parameters were set as follows: spray voltage, +3.5 kV or −3.0 kV; sheath gas flow, 40 arb; auxiliary gas flow, 10 arb; auxiliary gas temperature, 350 °C; capillary temperature, 320 °C. Complete data acquisition was performed in full scan/dd-MS^2^ (TOP10) mode. For full scan mode, resolution was 70,000 and scan range was 70–1050 mass/charge (*m*/*z*). For dd-MS^2^ mode, resolution was 17,500, and the top 10 intense ions were fragmented using stepped normalized collision energy at 20 and 40 eV.

QC samples were continuously injected multiple times before specimen analysis. All samples were then injected in random order, with QC samples detected every 10 specimens throughout the injection sequence.

### Data analysis of untargeted metabolomics

Raw data were imported into Compound Discoverer 3.1 software (Thermo Fisher Scientific, Waltham, MA, USA) for peak alignment, filtering, normalization, and identification. Pooled QC samples were used exclusively for compound identification. Metabolite identification parameters included mass tolerance of 5 parts per million and minimum peak intensity of 2,000,000. Compounds with coefficient of variation (CV) ≥ 30% in QC samples were filtered out. Ion features detected in blank samples with intensities > 30% of the average intensity in biological samples were considered background and were excluded from subsequent analyses. Metabolites were putatively annotated using the ChemSpider database by comparing theoretical and detected MS data, and the mzCloud database by matching referential and detected MS^2^ spectra [12]. Putative metabolites were selected based on mzCloud best match value of more than 80. Metabolite annotations were classified according to the Metabolomics Standards Initiative (MSI) guidelines [13], and all reported compounds were classified as level 2 (putatively annotated compounds). Subsequently, MetaboAnalyst 6.0 [14] was used for multivariate statistical analyses, including principal component analysis (PCA), partial least squares-discriminatory analysis (PLS-DA), and orthogonal partial least squares-discriminatory analysis (OPLS-DA) to discriminate comparison groups. For supervised models (PLS-DA and OPLS-DA), model robustness was performed using 5-fold cross-validation and 1000 permutation tests to assess over-fitting. Discriminative putative metabolites were selected based on variable importance in the projection (VIP) > 1.0 in OPLS-DA models and corrected *P* value <0.05 (Benjamini–Hochberg false discovery rate correction) in independent-samples *t* test. Hierarchical clustering analysis was conducted and visualized as heatmaps to illustrate putative metabolite concentration changes throughout the parasite life cycle. Pearson correlation coefficients were calculated to assess relationships among predominant putative metabolites. Random forest (RF) classification models were built to identify key signature putative metabolites, ranking the top 15 putative metabolites by mean decrease accuracy. Model performance was assessed by out-of-bag (OOB) error rate. Metabolite set enrichment analysis (MSEA) and pathway analysis were conducted using MetaboAnalyst 6.0 [14] to identify significant metabolic pathways involved in *A. cantonensis* development. Unless otherwise specified, statistical analyses and plotting were performed using R 4.2.2 software (R Foundation for Statistical Computing, Vienna, Austria).

## Results

### Multivariate and univariate statistical analysis of metabolite profiles

**Fig. 1:**
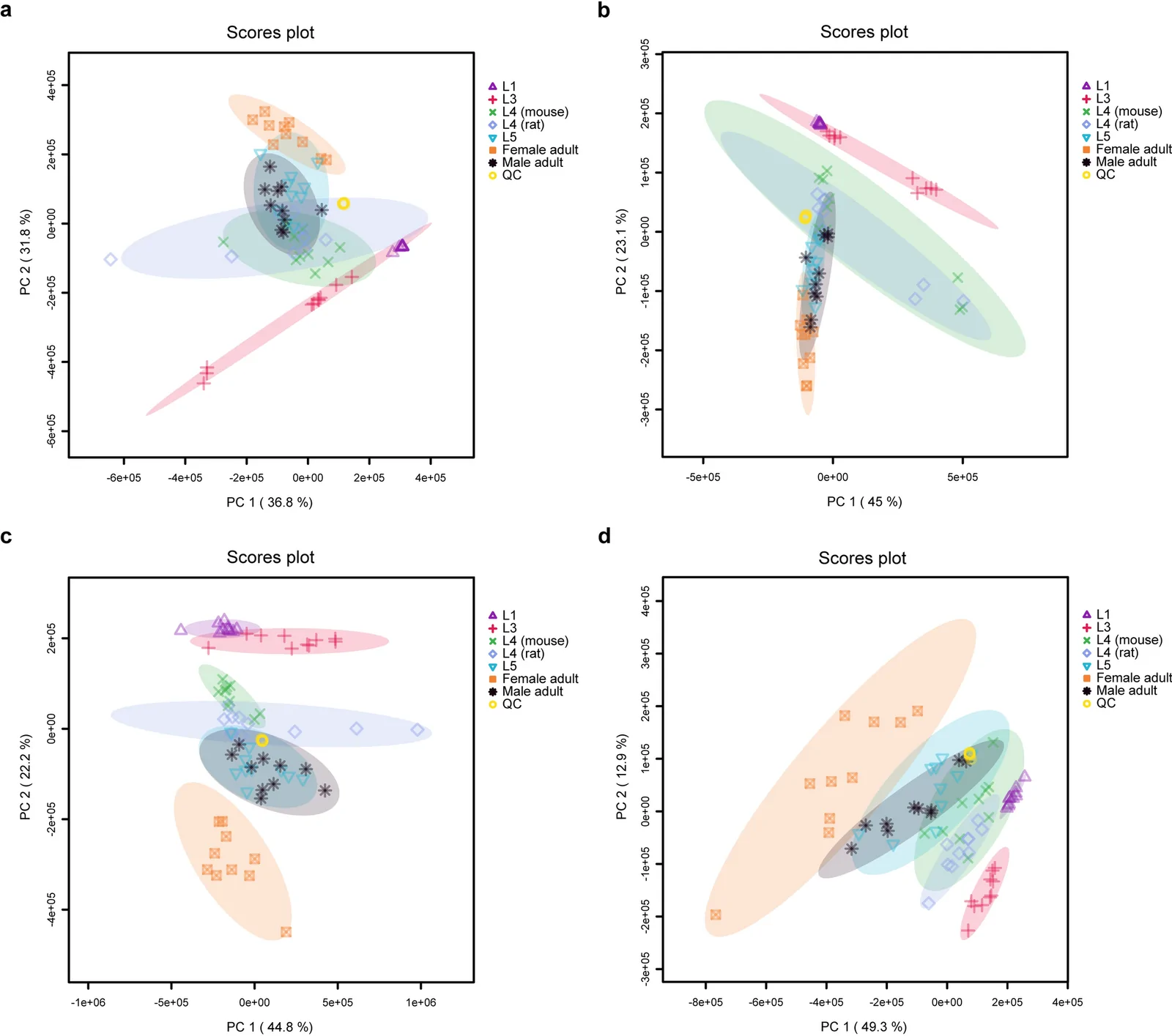
Two-dimensional score plots of principal component analysis from extracts of *Angiostrongylus cantonensis*. Each point represents an individual sample. **a** Aqueous extracts in positive electrospray ionization (ESI+) mode. **b** Aqueous extracts in negative electrospray ionization (ESI−) mode. **c** Organic extracts in ESI+ mode. **d** Organic extracts in ESI− mode

**Fig. 2:**
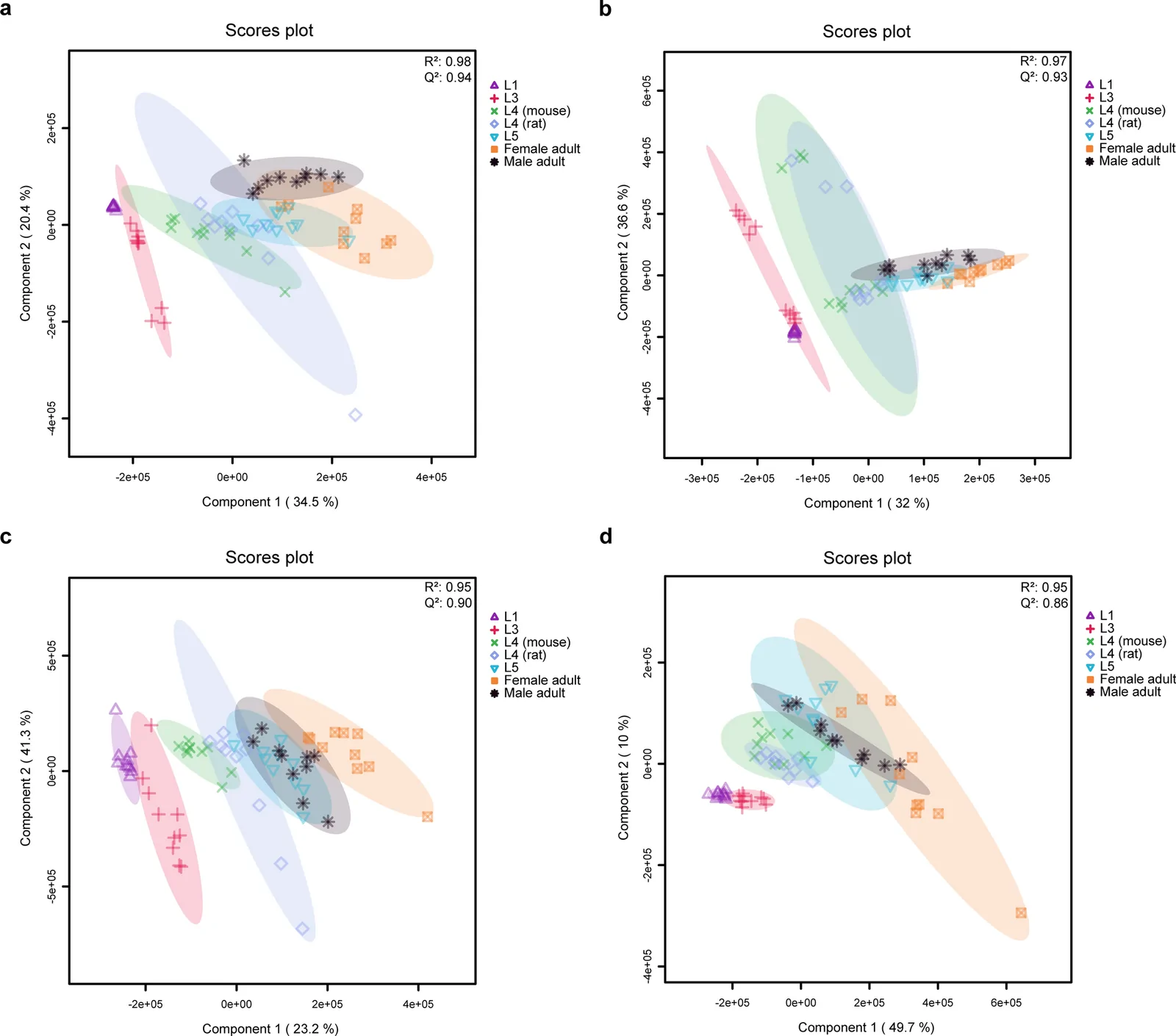
Two-dimensional score plots of partial least squares-discriminatory analysis from extracts of *Angiostrongylus cantonensis*. Each point represents an individual sample. **a** Aqueous extracts in positive electrospray ionization (ESI+) mode. **b** Aqueous extracts in negative electrospray ionization (ESI−) mode. **c** Organic extracts in ESI+ mode. **d** Organic extracts in ESI− mode

LC-MS analysis yielded 4517 ion features in ESI+ mode and 2613 in ESI− mode across different life cycle stages of *A. cantonensis*, respectively. Initially, PCA was performed to obtain a global overview of metabolic patterns without group considerations. As shown in Fig. 1, QC samples clustered tightly, confirming data stability and reproducibility. Based on the first two principal components, PCA revealed distinct separation between L4 and L3, and between male and female adults, in both ESI modes, indicating substantial metabolic differences between these groups. Partial overlap was observed between L3 and L1, between rat- and mouse-derived L4, and between male adults and L5 in both ion modes (Fig. 1). Supervised PLS-DA enhanced group separation, with all groups clearly differentiated by PLS1 and PLS2 in both ESI modes. High values of *R*^2^ and *Q*^2^ for each model confirmed data stability and good model fit (Fig. 2). Detailed model parameters, including cross-validation results and permutation test results, are provided in Additional file 2. All models exhibited *Q*^2^ > 0.5 and permutation *P* < 0.001, indicating no significant over-fitting. Thus, metabolic profiles of the worms changed dynamically during their development. OPLS-DA was then applied to maximize separation between pairwise comparisons and identify discriminant metabolic features using one predictive and one orthogonal component (Additional files 3–6). Complete separation between groups was achieved in both ESI modes, highlighting marked differences in metabolic phenotypes. High *R*^2^*Y* and *Q*^2^ values affirmed model stability, reliability, and predictive capacity (Additional file 7).

Univariate statistical analysis (adjusted *P* value < 0.05, fold change > 2.0) identified 1297 significant differential metabolic features between L3 and L1 (155 downregulated, 1142 upregulated), 2462 between mouse-derived L4 and L3 (319 downregulated, 2143 upregulated), and 2383 between rat-derived L4 and L3 (216 downregulated, 2167 upregulated). Between rat- and mouse-derived L4, 966 significant differential metabolic features (441 downregulated, 525 upregulated) were found. L5 versus rat-derived L4 showed 1042 differential features (49 downregulated, 993 upregulated). Compared with L5, 831 features were upregulated and 801 downregulated in female adults, whereas 414 were upregulated and 709 downregulated in male adults. A total of 1108 significant differential metabolic features (760 downregulated, 348 upregulated) were identified between male and female adults. These data also underscore distinct metabolic profiles, especially between L4 and L3, emphasizing metabolic shifts associated with definitive host entry (Additional file 8).

### Metabolites identification and analysis

**Fig. 3:**
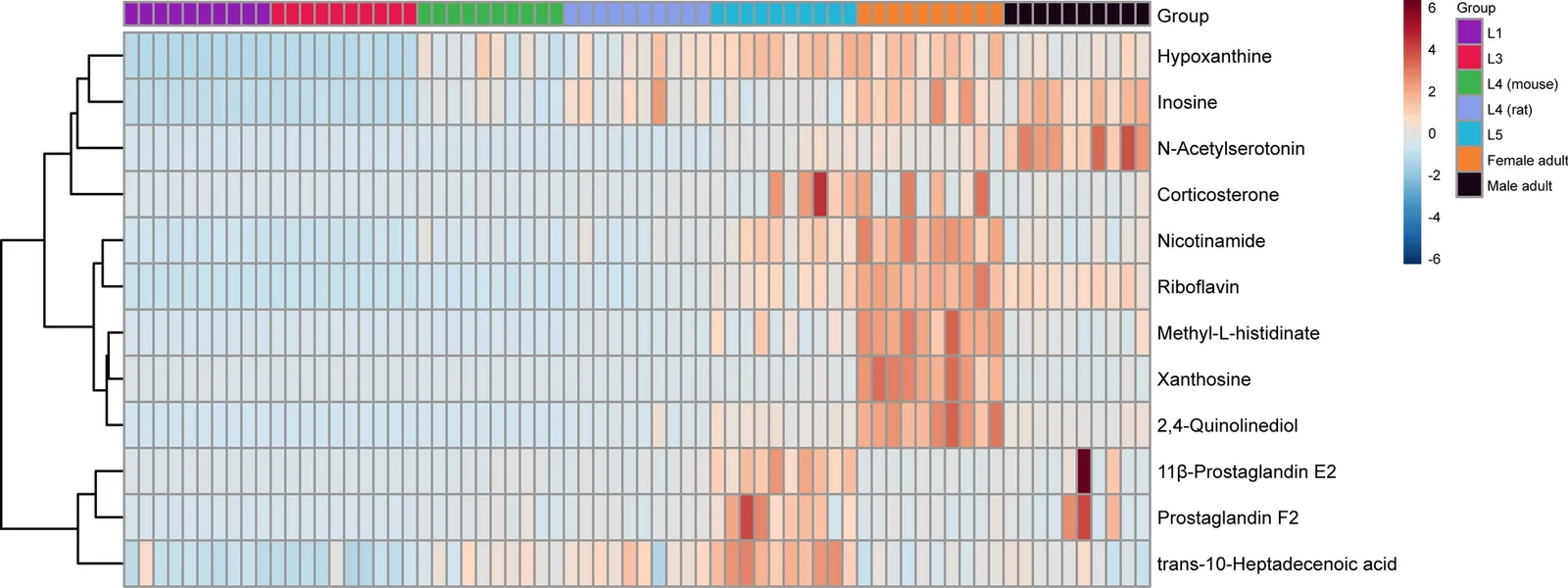
Heatmap visualization of significantly different predominant putative metabolites at various developmental stages of *Angiostrongylus cantonensis*. Each column represents a sample, and each row represents a putative metabolite. Scaled putative metabolite intensity is reflected by color key; red indicates higher intensity relative to mean, blue indicates lower intensity. Branch height represents similarity between putative metabolites, with shortest vertical distance indicating closest relationship

S-plots derived from OPLS-DA models, combined with VIP value > 1.0 and adjusted *P* value < 0.05 criteria, facilitated the selection of differential putatively annotated compounds. Database searching identified 127 unique level-II annotations in worms’ extracts, including fatty acyls, benzene and substituted derivatives, carboxylic acids and derivatives, purine nucleosides, organonitrogen compounds, and steroids and steroid derivatives. Detailed annotation information for each metabolic feature is provided in Additional file 9. As shown in Additional file 10, hierarchical cluster analysis demonstrated trends of significantly putative metabolites across all seven groups. Most putative metabolites were upregulated from the fourth larval stage onward. Twelve putative metabolites exhibiting differential abundance throughout the life cycle were selected for heatmap visualization. Hypoxanthine, inosine, nicotinamide, riboflavin, 11β-prostaglandin E2, and prostaglandin F2 increased at the fourth larval stage, whereas *N*-acetylserotonin, corticosterone, methyl-l-histidinate, and 2,4-quinolinediol were elevated at the fifth larval stage. Xanthosine was significantly upregulated in female adults, while trans-10-heptadecenoic acid peaked at the fifth larval stage but declined in adult stage (Fig. 3).

**Fig. 4:**
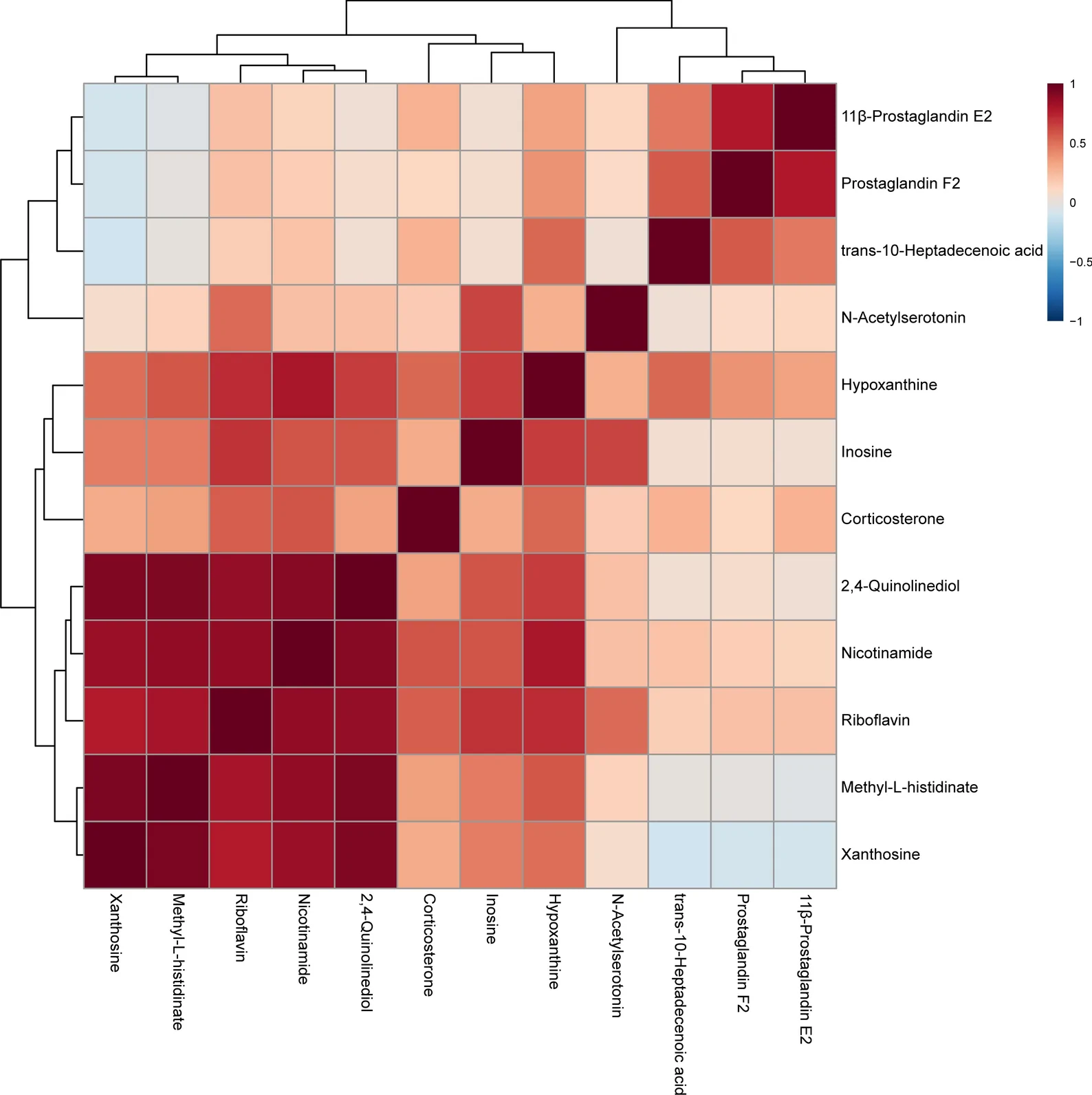
Correlation plot showing relationships among predominant putative metabolites across developmental stages of *Angiostrongylus cantonensis*. Correlation strength and direction are indicated by color intensity; red represents positive correlation, blue represents negative correlation, with darker color indicating stronger correlations

Correlation analysis revealed predominantly positive associations among major putative metabolites, suggesting coordinated regulation. Notably, 2,4-quinolinediol showed strong positive correlations with xanthosine (*r* = 0.92, *P* < 0.001), methyl-l-histidinate (*r* = 0.92, *P* < 0.001), nicotinamide (*r* = 0.90, *P* < 0.001), and riboflavin (*r* = 0.86, *P* < 0.001). Nicotinamide was positively associated with methyl-l-histidinate (*r* = 0.87, *P* < 0.001), riboflavin (*r* = 0.87, *P* < 0.001), xanthosine (*r* = 0.85, *P* < 0.001), and hypoxanthine (*r* = 0.80, *P* < 0.001). Methyl-l-histidinate correlated positively with xanthosine (*r* = 0.93, *P* < 0.001) and riboflavin (*r* = 0.81, *P* < 0.001) (Fig. 4). These clustering patterns were consistent with the heatmap.

**Fig. 5:**
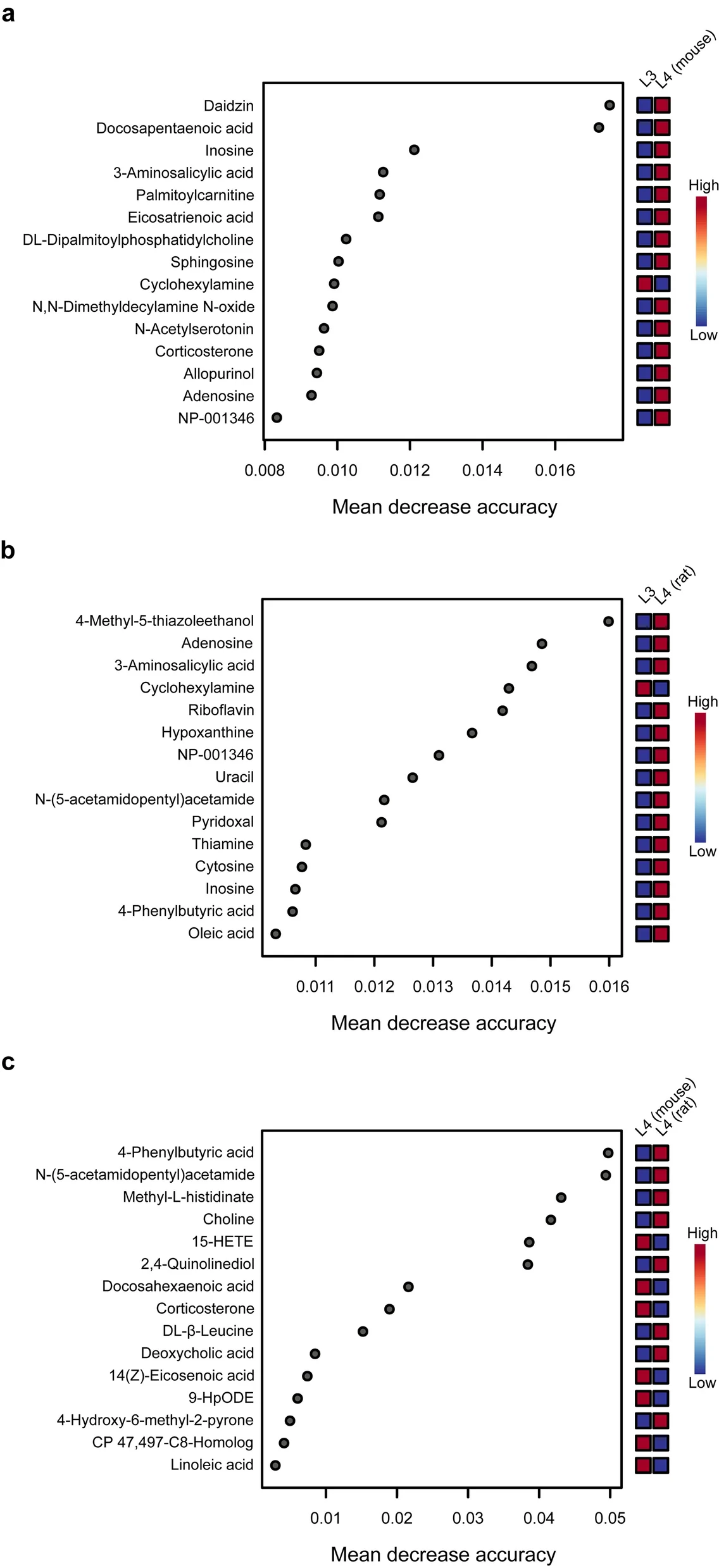
Random forest model classifying different developmental stages and hosts of *Angiostrongylus cantonensis* with important features. **a** Comparison between mouse-derived L4 and L3. **b** Comparison between rat-derived L4 and L3. **c** Comparison between rat-derived and mouse-derived L4. Features are ranked by mean decrease accuracy following permutation. Mini heatmap on right indicates intensity variations within groups; red indicates higher intensity, blue indicates lower intensity. *L3* third-stage larvae; *L4* fourth-stage larvae

Given that L4 develops in definitive hosts, with rat representing permissive host and mouse non-permissive host, RF models were constructed to distinguish L4 from L3 and L4 from different hosts based on putative metabolite abundance. The OOB error rates for the three RF models using 500 decision trees were all 0.0%, indicating strong classification performance (Additional file 11). Daidzin, docosapentaenoic acid (DPA), and 4-methyl-5-thiazoleethanol emerged as the most important variables discriminating L4 from L3, all upregulated in the former (Fig. 5a, b). Additionally, 4-phenylbutyric acid (4-PBA) and *N*-(5-acetamidopentyl)acetamide distinguished L4 from different hosts, with higher abundance in rat-derived L4 (Fig. 5c).

### MSEA and pathway analysis

**Fig. 6:**
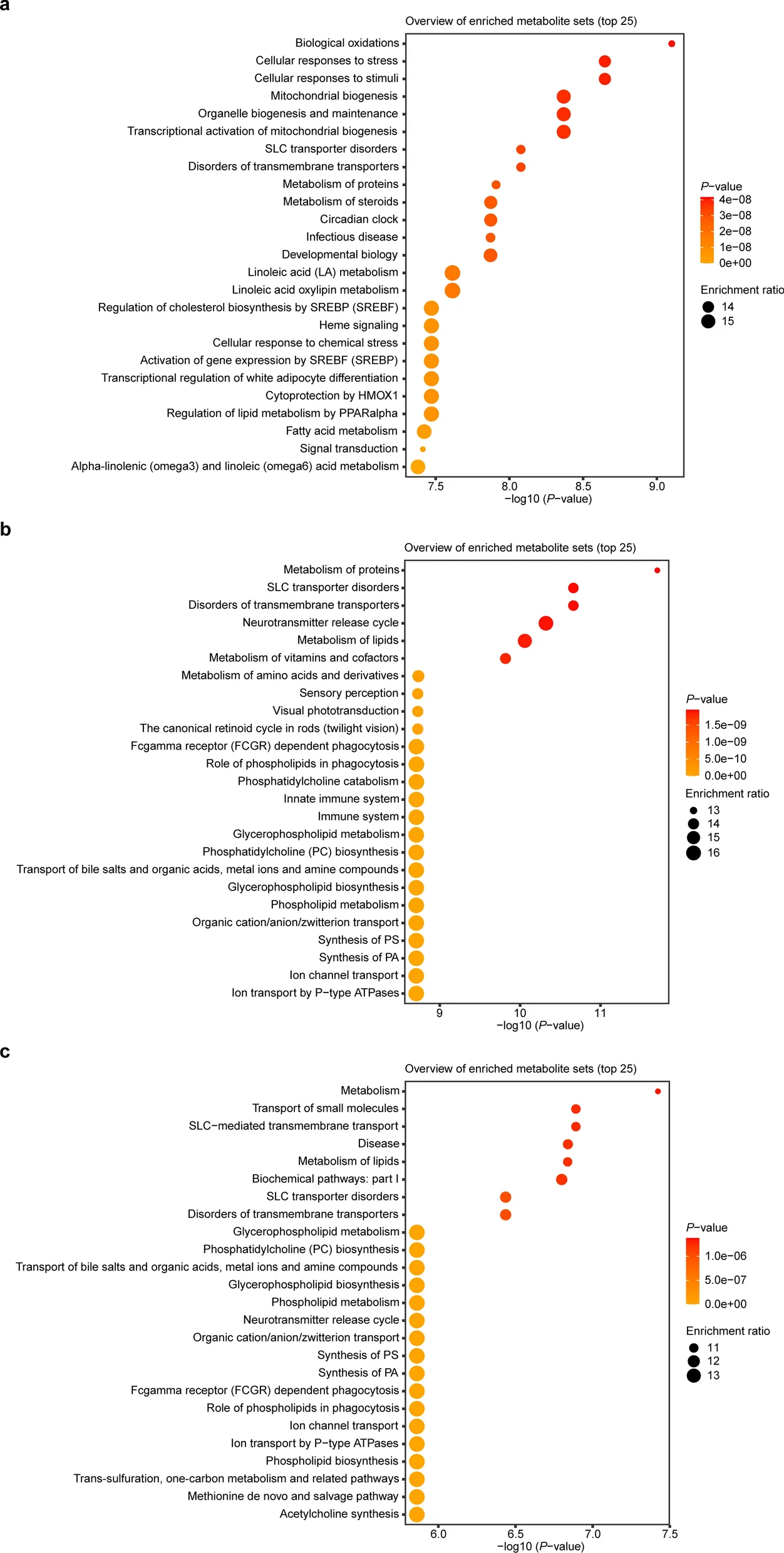
Summary plot for metabolite set enrichment analysis in extracts of *Angiostrongylus cantonensis*, ranked according to adjusted *P* value. **a** Comparison between mouse-derived L4 and L3. **b** Comparison between rat-derived L4 and L3. **c** Comparison between rat-derived and mouse-derived L4. Red to yellow gradient depicts *P* values from lowest to highest. Enriched metabolite sets are represented as nodes, with node size correlating with enrichment ratio. *L3* third-stage larvae; *L4* fourth-stage larvae

MSEA revealed distinct metabolite sets enriched between comparison groups. Comparison between mouse-derived L4 and L3 showed enrichment in cellular responses to stress, cellular responses to stimuli, mitochondrial biogenesis, organelle biogenesis and maintenance, and transcriptional activation of mitochondrial biogenesis, which exhibited high enrichment ratio and low *P* value (*P* < 0.001) (Fig. 6a). Enriched metabolite sets different between rat-derived L4 and L3 were SLC transporter disorders, disorders of transmembrane transporters, neurotransmitter release cycle, metabolism of lipids, and metabolism of vitamins and cofactors (*P* < 0.001) (Fig. 6b). In the comparison between rat- and mouse-derived L4, transport of small molecules, SLC-mediated transmembrane transport, disease, metabolism of lipids, and biochemical pathways were significantly enriched (*P* < 0.001) (Fig. 6c).

**Fig. 7:**
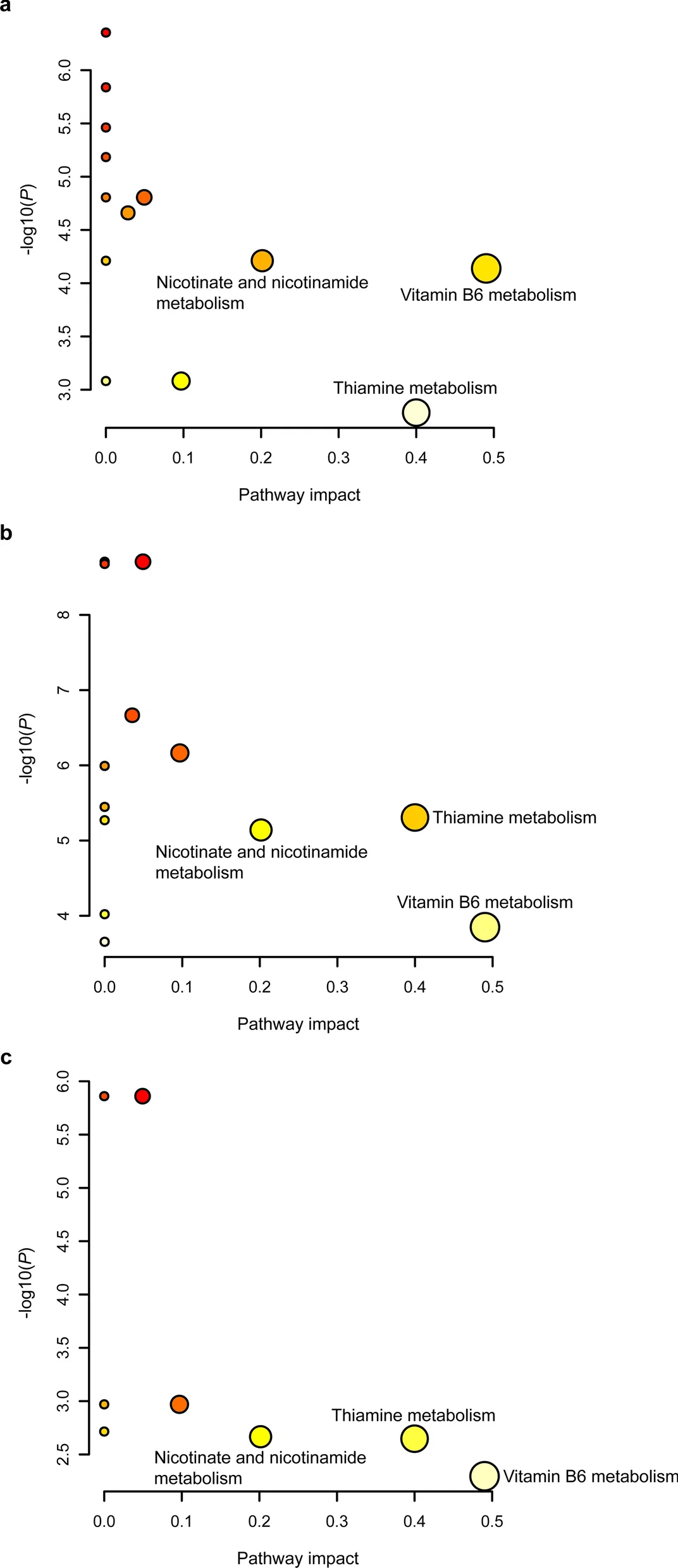
Pathway analysis of putative metabolites in *Angiostrongylus cantonensis* showing key nodes in metabolic pathways significantly altered during worm development. **a** Comparison between mouse-derived L4 and L3. **b** Comparison between rat-derived L4 and L3. **c** Comparison between rat-derived and mouse-derived L4. X-axis represents increasing metabolic pathway impact from pathway topology analysis; y-axis represents unadjusted *P* value from pathway enrichment analysis. Larger node sizes indicate higher pathway impact values; darker colors indicate lower *P* values. *L3* third-stage larvae; *L4* fourth-stage larvae

KEGG-based pathway analysis identified vitamin B6 metabolism, thiamine metabolism, and nicotinate and nicotinamide metabolism as significantly associated with L3-to-L4 development (impact factor > 0.2, *P* < 0.01) (Fig. 7a, b). Notably, these same three pathways were also markedly affected in L4 derived from different hosts (impact factor > 0.2, *P* < 0.01) (Fig. 7c), emphasizing their roles in both development and host interactions.

## Discussion

This study provides the comprehensive metabolomic characterization of *A. cantonensis* across its entire life cycle, from L1 to adults, revealing profound metabolic reprogramming associated with adaptation to diverse environments and host conditions. These findings confirm known biological transitions and reveal novel metabolic signatures that may serve as diagnostic or therapeutic targets for angiostrongyliasis. All functional interpretations discussed below are correlative and hypothesis-generating, requiring experimental validation through targeted functional studies.

The most pronounced metabolic shift occurred during the L3-to-L4 transition, a critical bottleneck marking parasite entry and establishment within the definitive host CNS. The upregulation of numerous putative metabolites in L4 reflects a massive anabolic shift likely required for growth, development, and host adaptation. Enrichment of cellular stress responses and mitochondrial biogenesis pathways aligns with coping with host immune responses and the brain parenchyma environment, consistent with findings in other parasitic nematodes and African trypanosome [15, 16]. Furthermore, the upregulation of purine (hypoxanthine) and purine nucleoside (inosine) in L4 is particularly noteworthy. Since many parasites lack de novo purine synthesis [17], heightened purine salvage pathways may reflect demand for nucleic acid precursors during rapid growth, as well as for ATP to support active metabolism. Based on previous reports, hypoxanthine and inosine possess immunomodulatory and protective properties [18], raising the hypothesis that their upregulation may help dampen host inflammatory responses to create a permissive environment, though this remains speculative and requires direct experimental testing. Concurrent upregulation of prostaglandins (11β-prostaglandin E2 and prostaglandin F2) in L4 further supports a possible immunomodulatory role. Prostaglandins are established mediators of inflammation and immune suppression, and parasite-derived prostaglandins represent a documented immune evasion strategy [19]. Elevated levels of these lipid mediators in L4 are consistent with active modulation of the host CNS immune environment to facilitate parasite survival and development, but this hypothesis awaits experimental validation.

A key finding is the metabolic distinction between L4 developing in permissive (rat) versus non-permissive (mouse) hosts, providing molecular evidence of host-shaped parasite physiology. The well-characterized chemical chaperone 4-PBA, which alleviates endoplasmic reticulum stress (ERS) by preventing misfolded protein aggregation and reducing the unfolded protein response [20], was markedly higher in rat-derived L4. We hypothesize that in the permissive host, 4-PBA upregulation may reflect active management of increased protein folding demands associated with rapid growth and adaptation, enabling proteostasis maintenance and avoidance of ERS-induced apoptosis. In contrast, lower 4-PBA levels in the non-permissive host may render parasites more susceptible to ERS, potentially contributing to developmental failure. This interpretation aligns with the concept that successful parasitism requires not only nutrient acquisition but also effective stress management [21], although direct evidence for this mechanism in *A. cantonensis* is currently lacking. Additionally, enrichment of pathways related to SLC transporter disorders and lipid metabolism in rat-derived L4 further underscores the potential importance of nutrient acquisition and transport for successful parasitism. Solute carrier proteins mediate transport of amino acids, sugars, nucleotides, and other substances across cell membranes [22]. Their differential regulation in rat-derived L4 is consistent with the hypothesis that rat provides a more favorable nutritional environment, prompting parasite metabolic machinery to upregulate transporters to capitalize on available resources. Lipid metabolism emerges as a central hub of host-associated adaptation [23], enrichment in rat-derived L4 may indicate active utilization of host lipids for energy production, membrane biosynthesis, and potentially signaling molecule synthesis. This aligns with the known dependence of many parasites on host-derived lipids due to limited de novo synthesis [24]. Upregulation of DPA, a long-chain polyunsaturated fatty acid, in L4 compared with L3 further underscores the potential importance of lipid remodeling during host adaptation. Polyunsaturated fatty acids are critical membrane components and precursors for bioactive lipid mediators, and their accumulation may reflect parasite preparation for the lipid-rich CNS environment [25].

Sexual dimorphism is clearly reflected in adult metabolic profiles. Female adults exhibit significantly higher xanthosine, a key purine metabolism intermediate and precursor for modified nucleotides in tRNAs in plants [26]. In nematodes, elevated xanthosine in female adults may reflect high nucleotide synthesis demand for egg production and reproductive activity. This aligns with observations in *Schistosoma mansoni*, where female reproductive biology is supported by specific metabolic pathways, including enhanced purine and NAD salvage pathways [27, 28]. Strong positive correlations among xanthosine, methyl-l-histidinate, 2,4-quinolinediol, and nicotinamide suggest coordinated regulation that may support oogenesis. Methyl-l-histidinate, a methylated histidine derivative, may play a role in reproductive processes [29]. Understanding these sex-specific metabolic pathways could reveal novel interventions that specifically impair female fertility or egg production, thereby blocking transmission without affecting the host.

Pathway analysis consistently highlighted vitamin B6 metabolism, thiamine metabolism, and nicotinate and nicotinamide metabolism as core hubs, significantly altered during the L3-to-L4 transition and between hosts. Vitamin B6 (pyridoxal phosphate) is a cofactor for numerous enzymes involved in amino acid metabolism, neurotransmitter synthesis, and glycogen degradation [30], its elevation during the fourth larval stage likely supports increased protein synthesis and metabolic flux required for growth. Thiamine (vitamin B1), as thiamine pyrophosphate, is essential for carbohydrate metabolism, particularly the pentose phosphate pathway and the citric acid cycle [31], its enrichment in L4 suggests a shift toward enhanced carbohydrate-derived energy production, consistent with increased metabolic demands. Nicotinate and nicotinamide metabolism generates NAD^+^ and NADP^+^, fundamental for redox balance, energy metabolism, and cellular signaling [32]. Nicotinamide itself has emerged as a key regulator of metabolism and aging, underscoring its biological significance [33]. The consistent modulation of these three vitamin-related pathways suggests they are core, non-redundant metabolic hubs essential for parasite survival and development. Targeting them with specific inhibitors could offer novel chemotherapeutic strategies, such as thiamine analogs have shown promise against *Plasmodium* and warrant exploration against *A. cantonensis* [34].

The identified putative metabolites and pathways have translational potential. RF models identified putative metabolite panels that accurately discriminate developmental stages and host sources, potentially enabling diagnostic biomarkers for angiostrongyliasis. For example, daidzin or DPA in cerebrospinal fluid could serve as a candidate biomarker for CNS invasion, pending clinical validation. Furthermore, the key pathways identified, including purine, vitamin, and lipid metabolism, represent promising drug targets. Parasitic helminths produce diverse unique metabolites with potential therapeutic applications, and exploring this metabolic diversity could yield new drug leads [35]. High-throughput screening of compound libraries against enzymes in these pathways could accelerate the discovery of new anthelmintic drugs, which are urgently needed due to limitations of current treatments.

Several limitations should be acknowledged. First, the untargeted nature of metabolomics and the lack of authentic standards restrict all compounds to putative annotation (MSI level 2). Despite solvent blank was analyzed and used for background subtraction, residual contamination cannot be entirely excluded for very low-abundance compounds. Therefore, all biological interpretations involving specific compounds should be considered provisional. Second, untargeted metabolomics is inherently semi-quantitative, many detected ion features remain uncharacterized. Third, metabolic profiles derived from whole-worm extracts represent averages across all tissues and cell types, obscuring tissue-specific metabolic signatures. Fourth, this observational study does not establish causality, all functional interpretations are hypothesis-generating and demand experimental testing through targeted approaches such as gene knockdown or knockout, pharmacological inhibition, or in vitro functional assays. Fifth, we used a single laboratory strain under controlled conditions, field isolates may differ. Finally, although the sample size is adequate, validation in larger, independent cohorts would strengthen generalizability.

## Conclusions

This study presents the comprehensive metabolomic characterization of *A. cantonensis* through its entire life cycle, encompassing key developmental transitions from L1 to adults in both intermediate and definitive hosts. By integrating LC-MS with rigorous multivariate and univariate statistical analyses, we have delineated the dynamic metabolic landscape that underpins parasite development, adaptation, and host interactions. The L3-to-L4 transition emerges as a critical metabolic bottleneck, characterized by extensive upregulation of purine metabolism, prostaglandin synthesis, and stress-response pathways. These changes are associated with the parasite’s shift from environmental survival to active establishment within the host CNS. Comparative analysis of L4 from permissive (rat) and non-permissive (mouse) hosts revealed distinct metabolic signatures, highlighting the profound influence of host environment on parasite physiology. The identification of host-associated putatively annotated metabolites, such as 4-PBA, provides novel molecular insights into host susceptibility and parasite fitness. Pathway analysis consistently implicated vitamin B6, thiamine, and nicotinate and nicotinamide metabolism as central hubs modulated throughout development and across different hosts. These pathways, essential for energy production, redox balance, and macromolecular synthesis, represent potential targets for chemotherapeutic intervention. Distinct metabolic profiles of male and female adults underscore sexual dimorphism in parasite metabolism, with implications for understanding reproductive biology.

In conclusion, this work enriches fundamental biological knowledge of *A. cantonensis* and provides a valuable resource for future research. The key putatively annotated metabolites and pathways identified offer promising leads for developing novel diagnostic biomarkers and discovering new drug targets aimed at disrupting parasite development and survival. The functional hypotheses generated here, while grounded in metabolomic associations and prior literature, require direct experimental validation. By bridging a critical knowledge gap in parasite metabolomics, this study establishes a foundation for translating metabolic insights into practical applications for disease control and environmental adaptation research.

## Supplementary Information

**Additional file 1.** Schematic workflow of the experimental design.

**Additional file 2.** **(a)** Cross-validation details of partial least squares-discriminatory analysis. The red star indicates the best classifier. **(b)** Permutation test results, indicating the separation distance.

**Additional file 3.** Orthogonal partial least squares-discriminatory analysis plots of aqueous extracts from *Angiostrongylus cantonensis* in positive electrospray ionization mode. Each point represents one sample in score plots.

**Additional file 4.** Orthogonal partial least squares-discriminatory analysis plots of aqueous extracts from *Angiostrongylus cantonensis* in negative electrospray ionization mode. Each point represents one sample in score plots.

**Additional file 5.** Orthogonal partial least squares-discriminatory analysis plots of organic extracts from *Angiostrongylus cantonensis* in positive electrospray ionization mode. Each point represents one sample in score plots.

**Additional file 6.** Orthogonal partial least squares-discriminatory analysis plots of organic extracts from *Angiostrongylus cantonensis* in negative electrospray ionization mode. Each point represents one sample in score plots.

**Additional file 7.** Permutation test results for models created by orthogonal partial least squares-discriminatory analysis.

**Additional file 8.** Volcano plots showing differences in metabolic feature abundance and statistical significance. Metabolic features with increased relative abundance are shown in red, decreased abundance in blue, and non-significant metabolic features in grey. FDR cutoff of 0.05 was applied to highlight statistically significant differences. *FDR* false discovery rate.

**Additional file 9.** Detailed annotation information for all 127 putatively annotated compounds. Background features detected in blank controls were filtered out prior to annotation; therefore, the listed compounds represent those that passed the blank-subtraction threshold.

**Additional file 10.** Heatmap visualization of significantly different putatively annotated compounds at various developmental stages of *Angiostrongylus cantonensis*. Each column represents a sample, and each row represents a putative metabolite. Scaled putative metabolite intensity is reflected by color key; red indicates higher intensity relative to mean, blue indicates lower intensity. Branch height represents similarity between putative metabolites, with shortest vertical distance indicating closest relationship.

**Additional file 11.** Random forest models showing cumulative error rates measured for each class. **(a)** Comparison between mouse-derived L4 and L3. **(b)** Comparison between rat-derived L4 and L3. **(c)** Comparison between rat-derived and mouse-derived L4. The overall error rate is shown as the red line, and other color lines represent the error rates for each class as indicated. *L3* third-stage larvae; *L4* fourth-stage larvae.

## Data Availability

Metabolomics data have been deposited in the EMBL-EBI MetaboLights database under accession number MTBLS14919 and MTBLS14959. All data supporting the findings of this study are available within the article and its supplementary files.

## Abbreviations

4-PBA: 4-Phenylbutyric acid
CNS: Central nervous system
CV: Coefficient of variation
DPA: Docosapentaenoic acid
dpi: Days post-infection
ERS: Endoplasmic reticulum stress
ESI: Electrospray ionization
GC-MS: Gas chromatography-mass spectrometry
IR: Infrared spectroscopy
L1: First-stage larvae
L3: Third-stage larvae
L4: Fourth-stage larvae
L5: Fifth-stage larvae
LC-MS: Liquid chromatography-mass spectrometry
MS: Mass spectrometry
MSEA: Metabolite set enrichment analysis
MSI: Metabolomics standards initiative
*m*/*z*: Mass/charge
NMR: Nuclear magnetic resonance
OOB: Out-of-bag
OPLS-DA: Orthogonal partial least squares-discriminatory analysis
PCA: Principal component analysis
PLS-DA: Partial least squares-discriminatory analysis
QC: Quality control
RF: Random forest
SD: Sprague Dawley
SLC: Solute carrier
VIP: Variable importance in the projection
