# Supplementary material for: Comprehensive metabolomic profiling of Angiostrongylus cantonensis across its life cycle reveals developmental and host-associated metabolic signatures
Source: Infect Dis Poverty. 2026 Sep 30;15:105. doi: 10.1186/s40249-026-01509-7 (PMC13625224; doi:10.1186/s40249-026-01509-7)
Supplement: Supplementary file 1 [file 40249_2026_1509_MOESM2_ESM.docx]

| Samples | Modes | Models | Perm R^2^Y | *P*-value | Perm Q^2^ | *P*-value |
| --- | --- | --- | --- | --- | --- | --- |
| Aqueous extracts | ESI+ | L1 vs. L3 | 0.99 | < 0.001 | 0.95 | < 0.001 |
|  |  | L3 vs. L4 (mouse) | 0.998 | < 0.001 | 0.951 | < 0.001 |
|  |  | L3 vs. L4 (rat) | 0.992 | < 0.001 | 0.964 | < 0.001 |
|  |  | L4 (mouse) vs. L4 (rat) | 0.994 | < 0.001 | 0.915 | < 0.001 |
|  |  | L4 (rat) vs. L5 | 0.937 | 0.001 | 0.868 | 0.001 |
|  |  | L5 vs. Female adult | 0.977 | < 0.001 | 0.95 | < 0.001 |
|  |  | L5 vs. Male adult | 0.966 | < 0.001 | 0.913 | < 0.001 |
|  |  | Female adult vs. Male adult | 0.973 | < 0.001 | 0.947 | < 0.001 |
|  | ESI- | L1 vs. L3 | 0.984 | < 0.001 | 0.924 | < 0.001 |
|  |  | L3 vs. L4 (mouse) | 0.992 | < 0.001 | 0.942 | < 0.001 |
|  |  | L3 vs. L4 (rat) | 0.98 | < 0.001 | 0.958 | < 0.001 |
|  |  | L4 (mouse) vs. L4 (rat) | 0.984 | < 0.001 | 0.915 | < 0.001 |
|  |  | L4 (rat) vs. L5 | 0.942 | < 0.001 | 0.862 | < 0.001 |
|  |  | L5 vs. Female adult | 0.982 | < 0.001 | 0.965 | < 0.001 |
|  |  | L5 vs. Male adult | 0.988 | < 0.001 | 0.942 | < 0.001 |
|  |  | Female adult vs. Male adult | 0.97 | < 0.001 | 0.946 | < 0.001 |
| Organic extracts | ESI+ | L1 vs. L3 | 0.917 | < 0.001 | 0.883 | < 0.001 |
|  |  | L3 vs. L4 (mouse) | 0.969 | < 0.001 | 0.958 | < 0.001 |
|  |  | L3 vs. L4 (rat) | 0.987 | < 0.001 | 0.969 | < 0.001 |
|  |  | L4 (mouse) vs. L4 (rat) | 0.987 | < 0.001 | 0.956 | < 0.001 |
|  |  | L4 (rat) vs. L5 | 0.882 | < 0.001 | 0.807 | < 0.001 |
|  |  | L5 vs. Female adult | 0.98 | < 0.001 | 0.951 | < 0.001 |
|  |  | L5 vs. Male adult | 0.992 | < 0.001 | 0.954 | < 0.001 |
|  |  | Female adult vs. Male adult | 0.978 | < 0.001 | 0.943 | < 0.001 |
|  | ESI- | L1 vs. L3 | 0.971 | < 0.001 | 0.946 | < 0.001 |
|  |  | L3 vs. L4 (mouse) | 0.99 | < 0.001 | 0.937 | < 0.001 |
|  |  | L3 vs. L4 (rat) | 0.971 | < 0.001 | 0.9 | < 0.001 |
|  |  | L4 (mouse) vs. L4 (rat) | 0.99 | < 0.001 | 0.852 | < 0.001 |
|  |  | L4 (rat) vs. L5 | 0.97 | < 0.001 | 0.787 | < 0.001 |
|  |  | L5 vs. Female adult | 0.987 | < 0.001 | 0.962 | < 0.001 |
|  |  | L5 vs. Male adult | 0.98 | < 0.001 | 0.938 | < 0.001 |
|  |  | Female adult vs. Male adult | 0.975 | < 0.001 | 0.933 | < 0.001 |

*ESI* electrospray ionization; *L1* first-stage larvae; *L3* third-stage larvae; *L4* fourth-stage larvae; *L5* fifth-stage larvae
